# Association of ABO Blood Type with Bleeding Severity in Patients with Acute Gastroesophageal Variceal Bleeding

**DOI:** 10.3390/medicina57121323

**Published:** 2021-12-03

**Authors:** Wei-Yu Lin, Ming-Yuan Hong, Chih-Hao Lin, Peng-Peng Chang, Shao-Chung Chu, Chia-Lung Kao

**Affiliations:** 1 Department of Emergency Medicine, National Cheng Kung University Hospital, College of Medicine, National Cheng Kung University, Tainan 70403, Taiwan; leafdancer1117@gmail.com (W.-Y.L.); myuan@mail2000.com.tw (M.-Y.H.); emergency.lin@gmail.com (C.-H.L.); yumi19881204@gmail.com (P.-P.C.); lioues@gmail.com (S.-C.C.); 2 Department of Emergency Medicine, Ministry of Health and Welfare Tainan Hospital, Tainan 70403, Taiwan

**Keywords:** ABO blood group system, gastroesophageal variceal bleeding, mortality

## Abstract

*Background and Objectives*: ABO blood types have been implicated as potential risk factors for various hemorrhagic diseases. No study has investigated the association between gastroesophageal variceal bleeding and ABO blood types. We aimed to evaluate the impact of ABO blood types on mortality and bleeding risk in acute gastroesophageal variceal bleeding. *Materials and Methods*: This is a retrospective observational study. Patients presenting with acute gastroesophageal varices bleeding diagnosed by endoscopy were enrolled, and were divided by blood type into a type O group and non-type O group. The outcomes were death within 30 days and the proportion of further bleeding. We used generalized linear mixed-effects models to analyze the outcomes. *Results*: A total of 327 patients and 648 records of emergency room visits were included. The 30-day mortality was 14.8% (21 of 142 patients) in the type O group, and 16.2% (30 of 185 patients) in the non-type O group (*p* = 0.532). Further bleeding within 30 days occurred in 34 cases (12.6%) in the type O group, and in 26 cases (6.9%) in the non-type O group (*p* = 0.539). *Conclusions*: There was no significant difference in blood transfusion volume in 24 h, recurrent bleeding rates, or mortality between patients with blood type O and those with non-type O.

## 1. Introduction

### 1.1. Background

Acute upper gastrointestinal (GI) bleeding is an important medical emergency, leading to significant morbidity and mortality. The overall annual incidence of upper GI bleeding ranges from 39 to 172 per 100,000 worldwide [1,2,3,4]. The most common causes of upper GI bleeding include peptic ulcer disease, followed by erosive disease and cirrhosis-associated gastroesophageal variceal bleeding.

Variceal bleeding is the major cause of death in cirrhotic patients [5]. It is also the major cause of GI bleeding in cirrhotic patients, and is responsible for approximately 70% of cases. The rebleeding rate for upper GI bleeding ranges from 7 to 16% [6]. The mortality of the first bleeding episode is estimated to be 15–20%. Furthermore, mortality varies according to the severity of liver cirrhosis: in the severe group (Child-Pugh C), it accounts for 30%, and as many as 70% of survivors have recurrent bleeding after a first variceal hemorrhage; in contrast, mortality is low in patients in the Child-Pugh A group [7,8]. Moreover, the one-year survival rate after variceal hemorrhage can be poor (32 to 80%) [9,10]. Other than the degree of liver dysfunction (Child-Pugh C versus Child-Pugh A–B), the size of the varices (large versus small varices) [11,12] and the appearance of the varices (red wale marks) [13,14,15] are known risks of variceal hemorrhage in patients with cirrhosis.

### 1.2. Objectives

The ABO blood group affects the severity, risk, and development of GI bleeding as a genetic factor. Recent studies have indicated that the ABO blood type has a profound influence on hemostasis. Individuals with blood type O have a longer bleeding time, and lower plasma levels of either factor VIII or von Willebrand factor (vWF) than individuals in with non-O blood types [16,17]; therefore, patients with type O blood have a relatively low incidence of venous thromboembolism compared to patients with non-type O blood [18]. Additionally, platelet function is compromised in the O blood group, which is also associated with a higher bleeding potency [19,20]. A number of studies have demonstrated a higher rate of bleeding complications in patients belonging to group O, including nonvariceal upper GI bleeding [21,22,23], postpartum bleeding [24], and severe traumatic bleeding [25].

In previous studies, it was found that blood type O has lower plasma levels of factor VIII or vWF [16,17], so blood type O is easier to bleed. However, coagulation in liver cirrhosis is more complicated because of thrombocytopenia, platelet dysfunction, and prolongation of the prothrombin time [26]. Studies have also found that patients with liver dysfunction will increase vWF to offset platelet dysfunction [26,27], and decreases in liver-derived anticoagulant factors, such as protein C, will offset the decrease in liver-derived procoagulant factors, such as factors V, VII, and X [28]. Hence, liver cirrhosis patients are in a state of platelet dysfunction, with prolonged prothrombin time, and relative hypercoagulation [29].

We therefore conducted this study to retrospectively evaluate the role of the ABO blood group system in gastroesophageal variceal bleeding and its risk factors.

## 2. Materials and Methods

### 2.1. Study Design and Setting

We enrolled all patients with an ICD diagnosis code of nontraumatic GI bleeding first, and then conducted a diagnosis of endoscopy-confirmed gastroesophageal varices who were admitted to the emergency room (ER) of National Cheng Kung University Hospital, a tertiary medical center in Tainan City, between 1 January 2014 and 31 August 2019, for a retrospective chart review study. Demographic data, medication history, the presentation of symptoms, systolic blood pressure, mean average pressure, pulse rate, ABO and Rh blood type, hemoglobin, platelet count, prothrombin time, and endoscopy findings were extracted from the chart. We excluded patients who were younger than 18 years of age, and patients with incomplete data.

### 2.2. Variables

According to Baveno VI consensus, it is recommended to use mortality as the primary endpoint, and additional endpoints should be reported, including treatment failure, need for salvage therapy, blood transfusion requirements, and days of ICU/hospital stay [30].

The primary outcome was death from any cause within 30 days after admission. The secondary outcomes included the receipt of endoscopic therapy at the first endoscopy, further bleeding (defined as persistent or recurrent bleeding), duration of the stay in the hospital, being cared for in the intensive care unit, the receipt of packed red blood cells (pRBCs) within the first 24 h, rubber band ligation, histoacryl injection, or the insertion of an Sengstaken-Blakemore (S-B) tube.

We defined further bleeding as a composite of persistent bleeding (i.e., bleeding that was not successfully controlled at the first endoscopy) or recurrent bleeding (i.e., bleeding that recurred after hemostatic treatment; hemostatic treatment included using a vasoactive drug for at least 24 h, rubber band ligation, histoacryl injection, or insertion of a S-B tube) which received secondary hemostatic treatment by endoscopy. Patients with overt signs of further bleeding after initial endoscopic control received an endoscopy together with further hemostatic treatment. The patients with recurrent bleeding that occurred after more than 30 days were counted as independent events.

### 2.3. Statistical Analysis

We stratified the groups into blood type O and type non-O. A *p* value less than 0.05 was considered statistically significant. We used *t*-tests and chi-squared tests to evaluate the patients’ baseline characteristics and clinical conditions at each patient’s visits. If the patients had multiple ER visits, we selected the latest visit’s data to represent the patients’ characteristics; a total of 327 patients were included.

We used chi-squared tests to analyze the proportions of whether the patient had been admitted to the ICU, and mortalities (including in-hospital mortality, mortality 30 days after discharge, and mortality 30 days since admission). We used a *t*-test to analyze the duration of admission according to each ER visit. Because the patient’s intervals at each visit were not independent events, considering the randomized effects of the patients, we used generalized linear mixed-effects models (GLMERs) with binomial families to analyze recurrent bleeding rates, including recurrent bleeding at 7 days and 30 days [31]. Because the blood transfusion volume was not independent of each visit, and considering a randomized effect model for the patients, we used GLMER with a binomial family to analyze the proportion of patients receiving blood transfusion, and the volume of transfused blood.

## 3. Results

### 3.1. Participants

**Figure 1:**
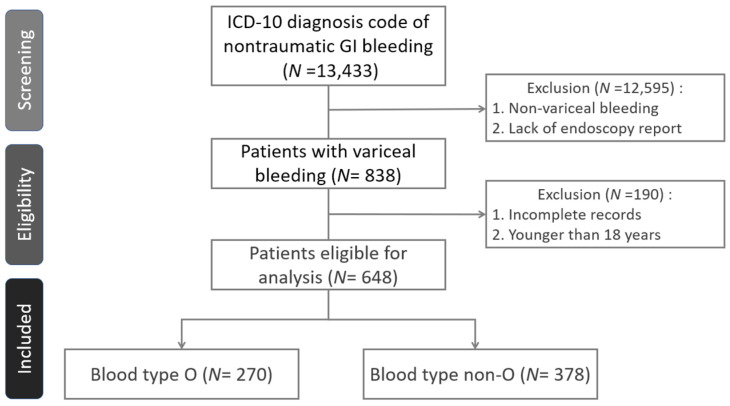
Flow diagram of case selection.

During the study period, a total of 13,433 ER visits with an ICD diagnosis code of nontraumatic GI bleeding underwent screening; 838 ER visits had gastroesophageal varices, and were confirmed by endoscopy. A total of 190 visits were excluded due to incomplete data or an age younger than 18 years; 327 eligible patients and 648 visits were enrolled in the analysis *(*Figure 1). All patients received a vasoactive drug on admission. The number of ER visits ranged from 1 to 9, and the corresponding patient numbers are shown in Table 1. A total of 142 patients were type O (43.4%), and 185 (56.6%) were non-O (A:82 (25.1%), B:84 (25.7%), AB:19 (5.8%)). All the patients had Rh positive antigen. The baseline demographic, clinical characteristics, and the endoscopic findings are shown in Table 2 and Table 3.

Patient characteristics were similar between the type O and non-type O groups, including average age, sex, initial hemoglobin level, platelet count, prothrombin time, systolic pressure, mean blood pressure, heart rate, coexisting diseases, and risk factors for bleeding (Table 3). The Child-Pugh score was not significantly different between the two groups (*p* = 0.845).

### 3.2. Endoscopic Findings and Treatment

Patients with blood type O had similar endoscopic findings as the patients with non-type O, including the proportion of the white nipple sign, the esophageal red color sign, or active bleeding (Table 4). Patients with blood type O had a similar proportion of initial treatments as the patients with non-type O, including gastroesophageal varices ligated by rubber bands, gastric varices that were injected with histoacryl, and poorly controlled bleeding that was treated with the insertion of an S-B tube.

Among the patients who had gastroesophageal variceal red color sign or active bleeding, a total of 18 cases did not receive initial treatment during the first endoscopy, 6 cases could not cooperate with or tolerate the procedure, 2 cases refused rubber ligation, 1 case had difficulty with ligation due to tumor obstruction, 4 cases had variceal bleeding with other nonvariceal bleeding for which the physicians did not perform rubber band ligation, and 5 cases had massive bleeding for which it was difficult to perform ligation, and for which the physicians inserted an S-B tube as initial management. All 18 patients received vasoactive drugs for at least 24 h, and a secondary endoscopy.

### 3.3. Primary and Secondary Outcomes

All-cause mortality at 30 days did not differ significantly between the two groups. A total of 21 patients (7.8%) were included in the type O group, and 30 patients (7.9%) were included in the type non-O group (Table 5). The patients with blood type O did not have significantly higher all-cause in-hospital mortality than the patients with non-type O (11.2% and 16.2%, respectively).

The duration of hospitalization did not differ between the type O and the type non-O groups (mean of 8.31 in the type O group, and 7.38 days in the type non-O group), and the two groups showed no significant difference in the percentage of patients who were admitted to the intensive care unit (7.8% and 8.9%), or the mean number of units of packed red cells received by transfusion (1.96 and 2.31 units, respectively).

Endoscopic hemostatic treatment was performed during the first endoscopy in 142 ER cases (52.6%) in the blood type O group, and in 202 ER cases (53.4%) in the non-type O group. Endoscopic rubber band ligation was performed in 124 cases (45.9%) in the blood type O group, and in 178 cases (47.1%) in the non-type O group. Histoacryl injection was performed in 17 cases (6.3%) in the type O group, and in 26 cases (6.9%) in the type non-O group. Eight patients (3.0%) in the blood type O group, and nine patients (2.4%) in the non-type O group had difficulty in bleeding control after endoscopic hemostatic treatment, and received S-B tube insertion.

Patients with blood type O had no significantly higher proportion of further variceal bleeding within 7 days after emergency department arrival, which was confirmed by secondary endoscopy (7.8% versus 4.2%, *p* value 0.408) (Table 5). Patients with blood type O had a higher percentage of further variceal bleeding within 30 days after initial treatment than patients with non-type O (12.6% versus 6.9%); however, there was no statistical significance after we adjusted the data by GLMER (*p* value 0.539). Among the 59 patients who had further gastroesophageal bleeding, 53 received rubber band ligation, and 10 received histoacryl injection.

## 4. Discussion

In this retrospective study involving patients with gastroesophageal variceal bleeding, we found that the blood type O group did not have higher mortality, including in-hospital mortality and mortality at 7 days or 30 days. A higher incidence of further bleeding after the initial endoscopic treatment was observed among patients in the blood type O group than among patients in the non-type O group. However, after we adopted GLMER to correct the calculation, the results showed no significant difference between the two groups. The incidence of further bleeding increased, but it did not reach statistical significance after we extended the observation period from 7 days to 30 days. The reason for this may be that the patients in the blood type O group had more bleeding events per patient. Twenty-one patients in the blood type O group had 34 recurrent bleeding events within 30 days; however, 23 patients in the non-type O group had 25 recurrent bleeding events within 30 days.

In hospitalized patients with upper gastrointestinal bleeding, increasing age, hemodynamic instability at presentation, and liver cirrhosis will increase the risk of in-hospital mortality [32]. Because of the higher risk of death, there are many studies exploring the prediction methods of esophageal varices [33,34,35]. In addition, band ligation is the main treatment of acute EV bleeding, but some patients are not suitable for ligation. Some studies have shown that endoscopic sclerotherapy or cyanoacrylate injection is helpful for the prognosis of these patients [36]. As the treatment method improves, it may also affect various factors, so more research is needed.

In one prospective case-control study that enrolled a total of 1033 patients with gastrointestinal bleeding, bleeders originating from the upper gastrointestinal tract showed that patients with type O blood had a higher risk of developing bleeding during the first 72 h of admission [23]. Among all-cause upper gastrointestinal bleeders, 15 patients with gastroesophageal varices (1.5%) were enrolled. This number of patients might not be enough to represent the characteristics of all patients with gastroesophageal variceal bleeding. Nonetheless, no other available article has investigated the association between gastroesophageal variceal bleeding and ABO blood types.

Our study had several limitations. First, although this retrospective study was conducted at National Cheng Kung University Hospital, a tertiary medical center in Tainan, Taiwan, which was the primary facility that conducted most of the endoscopic treatment in Tainan City, patients might have sought further evaluation or treatment at other hospitals during the following period of 30 days. Second, our study was a retrospective study with a limited sample size, and the risk of type I errors remained. Hence, more research is needed in the future. Third, we excluded patients who did not receive endoscopy, including patients with critical illness or terminal stage diseases. Fourth, because this is a retrospective study, the time of secondary endoscopy cannot fully meet the Baveno VI 5-day treatment failure. Thus, our results are not generalizable to patients with these conditions. Finally, the patients analyzed in our study were all Taiwanese, and it is unclear whether our findings apply to other ethnic groups.

## 5. Conclusions

In conclusion, in this retrospective study involving patients presenting with acute gastroesophageal varices that were confirmed and treated by endoscopy, there was no significant difference in the blood transfusion volume in 24 h, endoscopic treatment, 7-day or 30-day recurrent bleeding rates, or 7-day or 30-day mortality between patients with blood type O and those with non-type O. The patients with blood type O had no significantly higher risk of further bleeding than patients with non-type O.

Several studies have pointed out that blood types could affect blood coagulation function. Gastroesophageal varices bleeding is one of the main reasons patients with liver cirrhosis go to the emergency department. This is the first article to analyze the influence of blood types on gastroesophageal varices bleeding. Although this was a retrospective study, ABO blood type did not significantly affect the volume of blood transfusion within the first 24 h, or the rebleeding rate or mortality within 30 days.

## Figures and Tables

**Table 1:** Number of emergency room visits.

Number of ER Visits	Total Patient Number (*N* = 327)
1	179
2	69
3	36
4	20
5	10
6	4
7	5
8	2
9	2

**Table 2:** Baseline characteristics of the patients’ visiting.

Total Patient Number	Total (*N* = 327)	Type O (*N* = 142)	Type Non-O (*N* = 185)	*p* Value
Characteristics				
Age mean ± SD—years	63.09 ± 15.12	64.2 ± 12.30	65.1 ± 13.39	0.516
Male sex–no. (%)	219 (67.0)	98 (69.0)	121 (65.4)	0.569
Coexisting diseases–no. (%)				
Hypertension (%)	77 (23.5)	31 (21.8)	46 (24.9)	0.611
Diabetes mellitus (%)	112 (34.3)	53 (37.3)	59 (31.9)	0.422
End-stage renal disease with hemodialysis (%)	22 (6.7)	11 (7.7)	11 (5.9)	*
Hepatoma (%)	127 (38.8)	49 (34.5)	78 (42.2)	0.196
Total Patient ER visits	Total (*N* = 648)	Type O (*N* = 270)	Type non-O (*N* = 378)	*p* Value
Initial data on admission				
Hemoglobin level ± SD—g/dL	8.83 ± 2.61	8.98 ± 2.45	8.73 ± 2.24	0.271
Platelet count ± SD—10^3^/µL	120 ± 79.24	124.88 ± 92.73	116.58 ± 74.19	0.613
Prothrombin time ± SD—seconds	16.1 ± 4.40 (1.46 ± 0.38)	16.12 ± 4.33 (1.47 ± 0.42)	16.08 ± 5.16 (1.45 ± 0.39)	0.598 (0.926)
Systolic blood pressure ± SD—mmHg	125.2 ± 28.6	127.0 ± 25.6	124.0 ± 25.1	0.123
Systolic blood pressure < 90 mmHg–no. (%)	96 (14.8)	38 (14.1)	58 (15.3)	0.705
Mean blood pressure ± SD—mmHg	90.28 ± 20.5	91.9 ± 19.1	89.1 ± 17.9	0.072
Mean blood pressure < 65 mmHg–no. (%)	42 (6.5)	20 (7.4)	22 (5.8)	0.420
Heart rate–beats/min	98.16 (23.47)	97.92	98.32	0.859
Heart rate > 100 beats/min–no. (%)	306 (47.2)	133 (49.3)	173 (45.8)	0.445
Child-Pugh Score (Level)–no. (%)				0.845
5 to 6 (A)	223 (36.0)	89 (32.9)	146 (38.6)	
7 to 9 (B)	298 (46.0)	122 (45.3)	174 (46.1)	
10 to 15 (C)	117 (18.1)	59 (21.9)	58 (15.3)	
Risk factors for bleeding				
Aspirin or clopidogrel use (%)	14 (2.2)	7 (2.6)	7 (1.9)	*
Warfarin or direct oral anticoagulant use (%)	3 (0.5)	2 (0.7)	1 (0.3)	*

* The ratio was less than 10%; *p* value was not calculated. Plus–minus values are the means ± SDs. Percentages may not total 100 because of rounding.

**Table 3:** Endoscopic findings and treatment.

Characteristics	Total (*N* = 648)	Type O (*N* = 270)	Type Non-O (*N* = 378)	*p* Value
Gastroesophageal varices appearance				
White nipple sign (%)	23 (3.5)	10 (3.7)	13 (3.4)	*
Red color sign, active bleeding (%)	299 (46.1)	123 (45.6)	176 (46.6)	0.459
Endoscopic treatment				
Rubber ligation (%)	302 (46.6)	124 (45.9)	178 (47.1)	0.668
Histoacryl injection (%)	43 (6.6)	17 (6.3)	26 (6.9)	*
S-B tube insertion (%)	17 (2.6)	8 (3.0)	9 (2.4)	*

* The ratio was less than 10%; *p* value was not calculated.

**Table 4:** Primary Outcomes-Mortality.

Mortality	Total (*N* = 327)	Type O (*N* = 142)	Type Non-O (*N* = 185)	*p* Value
All-cause in-hospital mortality–no. (%)	46 (14.1)	16 (11.2)	30 (16.2)	0.265
All-cause 7-day mortality–no. (%)	13 (4.0)	4 (2.8)	9 (4.9)	0.306
All-cause 30-day mortality–no. (%)	51 (15.6)	21 (14.8)	30 (16.2)	0.532

**Table 5:** Secondary outcomes.

	Total (*N* = 648)	Type O (*N* = 270)	Type Non-O (*N* = 378)	*p* Value
ICU admission–no. (%)	52 (8)	21 (7.8)	31 (8.9)	0.857
Admission days ± SD	8.37 ± 7.2	8.31 ± 11.68	7.38 ± 7.52	0.832
Further bleeding				
Further bleeding confirmed by PES in 7 days (%)	37 (5.7)	21 (7.8)	16 (4.2)	0.408
Further bleeding confirmed by PES in 30 days (%)	59 (9.1)	34 (12.6)	25 (6.9)	0.539
Pack RBC transfusion amount	2.16 ± 2.37	1.96 ± 2.25	2.31 ± 2.26	0.071

Plus–minus values are the means ± SDs. Percentages may not total 100 because of rounding.

## Data Availability

The data in this study are available from the corresponding author upon reasonable request.
